# Emerging Role of the Calcium-Activated, Small Conductance, SK3 K^+^ Channel in Distal Tubule Function: Regulation by TRPV4

**DOI:** 10.1371/journal.pone.0095149

**Published:** 2014-04-24

**Authors:** Jonathan Berrout, Mykola Mamenko, Oleg L. Zaika, Lihe Chen, Wenzheng Zang, Oleh Pochynyuk, Roger G. O'Neil

**Affiliations:** 1 Department of Integrative Biology, The University of Texas Health Science Center Medical School, Houston, Texas, United States of America; 2 Department of Internal Medicine-Division of Renal Diseases and Hypertension, The University of Texas Health Science Center Medical School, Houston, Texas, United States of America; University of Pittsburgh, School of Medicine, United States of America

## Abstract

The Ca^2+^-activated, maxi-K (BK) K^+^ channel, with low Ca^2+^-binding affinity, is expressed in the distal tubule of the nephron and contributes to flow-dependent K^+^ secretion. In the present study we demonstrate that the Ca^2+^-activated, SK3 (K_Ca_2.3) K^+^ channel, with high Ca^2+^-binding affinity, is also expressed in the mouse kidney (RT-PCR, immunoblots). Immunohistochemical evaluations using tubule specific markers demonstrate significant expression of SK3 in the distal tubule and the entire collecting duct system, including the connecting tubule (CNT) and cortical collecting duct (CCD). In CNT and CCD, main sites for K^+^ secretion, the highest levels of expression were along the apical (luminal) cell membranes, including for both principal cells (PCs) and intercalated cells (ICs), posturing the channel for Ca^2+^-dependent K^+^ secretion. Fluorescent assessment of cell membrane potential in native, split-opened CCD, demonstrated that selective activation of the Ca^2+^-permeable TRPV4 channel, thereby inducing Ca^2+^ influx and elevating intracellular Ca^2+^ levels, activated both the SK3 channel and the BK channel leading to hyperpolarization of the cell membrane. The hyperpolarization response was decreased to a similar extent by either inhibition of SK3 channel with the selective SK antagonist, apamin, or by inhibition of the BK channel with the selective antagonist, iberiotoxin (IbTX). Addition of both inhibitors produced a further depolarization, indicating cooperative effects of the two channels on Vm. It is concluded that SK3 is functionally expressed in the distal nephron and collecting ducts where induction of TRPV4-mediated Ca^2+^ influx, leading to elevated intracellular Ca^2+^ levels, activates this high Ca^2+^-affinity K^+^ channel. Further, with sites of expression localized to the apical cell membrane, especially in the CNT and CCD, SK3 is poised to be a key pathway for Ca^2+^-dependent regulation of membrane potential and K^+^ secretion.

## Introduction

Calcium-activated potassium channels, K_Ca_, are a small group of potassium channels that are widely expressed in numerous tissues ranging from neurons to vascular endothelial cells [1]–[5]. As with other K^+^ channels, the K_Ca_ channels can play a major role in regulating the plasma membrane electrical potential difference, Vm. However, their classical regulation by intracellular Ca^2+^, [Ca^2+^]_i_, leads to a highly dynamic coupling between Vm and [Ca^2+^]_i_ which appears to underlie their central role in a wide array of functions ranging from neuronal excitability [6], [7], to modulation of vascular smooth muscle tone [8], [9], to cell volume regulation [10], [11]. Indeed, depending on the types of K_Ca_ channels expressed by a particular cell type, the hyperpolarization of the cell membrane following Ca^2+^-induced activation of a given K_Ca_ channel can either enhance Ca^2+^ influx through non-voltage-activated, Ca^2+^-permeable channels, such as TRP channels, or reduce Ca^2+^ influx in the case of voltage-activated Ca^2+^ channels [4], [12].

To date, five subtypes of Ca^2+^-activated K^+^ channels have been identified: the large-conductance channel (BK, K_Ca_1.1), the intermediate-conductance channel (IK1, KCa3.1), and three small-conductance channels (SK1, K_Ca_2.1; SK2, K_Ca_2.2; and SK3, K_Ca_2.3) [1]–[3]. While the channels have similar structure (6–7 transmembrane segments, a pore loop region, and assembly as homo/heterotetramers), the gating mechanisms can differ, especially between BK and the other channels. Indeed, BK is gated by both membrane potential (activates with depoloarization) and intracellular Ca^2+^. Further, the Ca^2+^ binding sites in the C-terminus, the “Ca^2+^ bowl,” of the channel-forming α-subunit of BK are characterized with a low Ca^2+^ binding affinity requiring high cytoplasmic levels of Ca^2+^ for activation (EC_50_ = 1–11 µM; [13]–[15]); however, the Ca^2+^ affinity can be modulated by binding of selective BK β subunits. In contrast, IK and SK channels are voltage insensitive. However, the IK/SK Ca^2+^ binding site is the ubiquitous Ca^2+^-sensor, calmodulin, constitutively bound to the C-terminus of the channel, which is characterized by a high Ca^2+^ binding affinity with a Ca^2+^ EC_50_ for gating near 300–600 nM [16]–[18]. As a consequence, the SK channels are highly sensitive “Ca^2+^ sensors” intimately linking [Ca^2+^]_i_ to membrane potential and K^+^ efflux in all cells where these channels are expressed.

In the mammalian kidney, K^+^ channels expressed at the luminal (apical) membrane of the late distal tubule and cortical collecting duct (CCD) are Ba^2+^-sensitive (blocker) channels that represent the dominant conductance of the apical membrane (see [19], [20]). Hence, the underlying channels serve as key K^+^ secretory pathways which regulate K^+^ excretion and, hence, K^+^ homeostatis [21]–[24]. It has been shown that the ROMK channel (K_ir_1.1), an inward rectifier K^+^ channel from the K_ir_ family, is the resting, Ba^2+^-sensitive, channel responsible for K^+^ secretion under normal physiological conditions [5], [25]–[27]. Under stimulated states, however, it is becoming apparent that other K^+^ channels can contribute to K^+^ secretion. Indeed, it has been shown that elevated flow rates to the late distal tubule or the CCD leads to enhanced K^+^ secretion via activation of the luminal BK channel giving rise to the phenomenon of flow-dependent K^+^ secretion [24], [28], [29]. This is a Ca^2+^-dependent process [28], [30]–[32] that we and others have shown is paralleled by flow-induced Ca^2+^ influx arising from activation of the Ca^2+^-permeable TRPV4 channel, a noted mechanotransducer channel [33]–[36], that is highly expressed in the renal collecting duct cells [28], [31], [32]. However, whether the BK channel can fully account for the flow-induced K^+^ secretion or during other Ca^2+^-dependent K^+^-secretory stimulatory states is not known. Indeed, while a major fraction of the enhanced flow-induced K^+^ secretion is abolished in various animals models deficient in BK or certain regulatory β subunits [37]–[39], it is also known that the enhanced flow will stimulate luminal ATP release from distal tubule cells into the lumen [40], [41] and, in turn, inhibit ROMK channel activity [42]. What channel accounts for the continued relatively high levels of K^+^ secretion under these conditions is currently not known. However, we have recently demonstrated that mouse M-1 collecting duct cells express both the BK channel and the SK3 channel where both channels are activated by mechanical stimulation in a Ca^2+^-dependent manner [43]. With the high Ca^2+^ binding affinity of SK3, especially over BK, we speculate that the SK3 channel is an “early effector” that would respond to modest elevations in [Ca^2+^]_i_ during K^+^ secretory stimulatory events where the channel would function as an important pathway contributing to regulation of Vm, K^+^ secretion and K^+^ homeostasis.

The goal of this study was to determine if the SK3 channel was expressed in the late distal tubule and other nephron segments of the kidney and, if so, was it functionally regulated by Ca^2+^ influx. We found that SK3 is expressed in the mouse kidney with immunohistochemical staining showing apparent strong expression in the thick ascending limb, the distal convoluted tubule, and the entire collecting duct systems, including the connecting tubule (CNT) and the cortical collecting duct (CCD). In the CCD SK3 was shown to be expressed in both principal cells (PC) and intercalated cells (IC), with pronounced expression along the apical (luminal) border and subapical regions. Selective activation of TRPV4, leading to Ca^2+^ influx, led to cell hyperpolarization that was partially inhibited by application of either apamin, a selective SK channel inhibitor, or iberiotoxin (IbTX), a selective BK channel inhibitor. These findings demonstrate that functional SK3 channels are expressed in the late distal tubule/collecting duct and that they are regulated by TRPV4-mediated Ca^2+^ influx where they would appear to play a key role in regulating membrane potential and K^+^ secretion in the TRPV4-positive cells of the CNT and CCD.

## Materials and Methods

C57BL/6 mice were maintained on a normal diet with free access to water. Kidneys were removed and used for experimentation as outlined for each protocol below. All studies were carried out in strict accordance with recommendations in the Guide for the Care and Use of Laboratory Animals of the NIH. All animal protocols were approved by the Institute for Animal Care and Use Committee of The University of Texas Health Science Center (AWA#: A3414-01).

### Kidney RT-PCR

**Figure 1:**
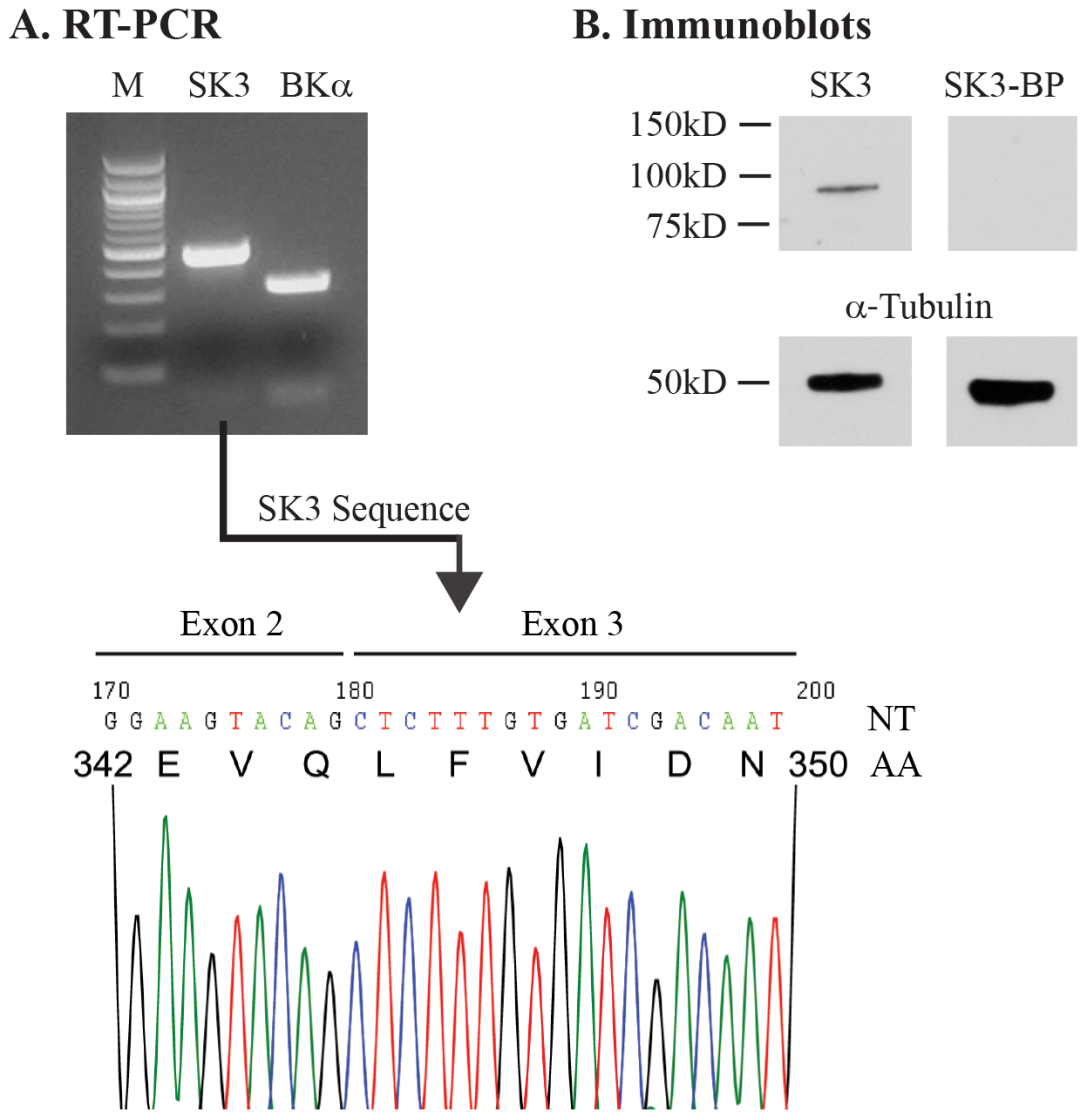
SK3 expression in WT mouse kidney. **A.** RT-PCR analysis using whole kidney mRNA extracts revealed prominent bands of the appropriate size on agarose gels for both SK3 (473 bp) and BKα (318 bp), demonstrating expression of both of these channels in the kidney. SK3 primers were selected to cross the exon 2 and exon 3 borders to rule out amplification of intron sequences from genomic DNA. The electropherogram for SK3 is shown with both nucleotide sequences (NT) and amino acid sequences (AA) indicated for the segment across the exon border region, demonstrating that the PCR product does not originate from genomic DNA. 100-bp marker standards are shown (Lane M). **B.** Western blot of WT mouse kidney-SK3. SK3 protein is expressed as a single band near 90 kD in mouse kidney. SK3 blocking peptide (SK3-BP) was used as a control to verify antibody specificity which, as shown, abolished binding of the anti-SK3 antibody (right lane). Alpha-tubulin expression was used as a loading control (lower panel).

Total RNAs were prepared from whole kidney using TRIzol reagents (Invitrogen) following the manufacturer's instruction as described previously [44], [45]. All RNA samples were pretreated with DNase I to eliminate potential genomic contamination. RT-PCR products were verified in separate reactions in which the reverse transcriptase was omitted (data not shown). The first strand cDNA Synthesis kit (Roche) was used to synthesize all cDNAs. PCR was performed using specific primers for SK3 (KCNN3) (forward: 5′-GCCCTGTTTGAAAAGAGAAAGCGAC-3′ and reverse: 5′-GCATCAGTGAAGAGTTTGCTATGGAGC-3′). SK3 primers were selected to cross the boundary been exon 2 and 3 to rule out products derived from genomic DNA. Nucleotide sequencing verified that the PCR product was derived from SK3 mRNA (see Figure 1A). For BKα (KCNMA1), standard primers were selected for variant 1 (forward: 5′-CCTCTTCATCATCTTGCTCTGGCG-3′ and reverse: 5′-TGGCAGGATTCTATTGGGTTTGACG-3′ as typically done. PCR cycling included 35 cycles: denaturation at 95°C for 20 s, primer annealing at 55°C for 30 s, and extension for 1 min at 72°C, followed by a 10 min completion step at 72°C. All PCR products were verified by agarose gel analyses (1% agarose, 0.5 mg/ml ethidium bromide) against 100-bp standard markers (New England Biolabs).

### Western blotting

In preparation for Western blotting, mice were anesthetized with isoflurane inhalation and the kidneys dissected free and immediately processed for immunoblotting. Briefly, kidneys were immediately sliced into several small pieces, on ice. Tissue was then homogenized with 4× volume of ice-cold lysis buffer (50 mM Tris, 1% Triton X-100, 5 mM EDTA, pH 7.5), containing protease inhibitor cocktail (1 ml/20 g of tissue, Sigma-Aldrich). The homogenates were then immediately centrifuged at 15000 g for 15 min at 4°C and the supernatants collected and stored at −20°C until use.

For Western blots, 5× Laemmli buffer was added to sample protein and then heated for 10 min at 70°C. Next, 20 µg of sample protein was run in a 4–15% SDS-PAGE gradient gel, transferred to PVDF membrane, and blocked with 5% nonfat milk for 1.5 hrs at RT. Membranes were incubated with a well-characterized, high specificity, anti-SK3 primary antibody (anti-K_Ca_2.3 directed against the N-terminus, 1∶100, Alomone Cat. #APC-025; see references [46]–[48] and Alomone web site) overnight at 4°C. After washing, membranes were incubated with secondary antibody (anti-rabbit, 1∶1000, Invitrogen). Alpha-tubulin (≈50 kD) was used as a loading control (monoclonal anti-α-tubulin, 1∶1000, Sigma). An SK3 specific blocking peptide (Alomone) was used to verify specificity of the SK3 primary antibody.

### Immunohistochemistry

Standard immunocytochemistry procedures were used to prepare and immunostain kidney tissue as previously described [32], [43]. Mice were anesthetized with isoflurane inhalation and the kidneys then fixed by cardiac perfusion with 40 ml of ice cold fixative solution (4% paraformaldehyde in 0.1% cacodylate buffer, pH 7.4). Kidneys were then removed and post-fixed in fixative solution for an additional 24 hrs, at 4°C. Following fixation, the tissue was placed in a 30% sucrose PBS for 48–72 hrs at 4°C. Fixed kidneys were then frozen at −20°C and sectioned (5 µm thick, sagittal and transverse sections) with use of an OTF 5000 cryostat (Bright Instruments).

Prior to staining, tissue sections were allowed to warm to room temperature, and washed with 1× TBS. Subsequently, kidney sections were incubated with 0.25% Triton X-100, again washed with TBS, and then blocked by incubation with 5% donkey serum. The tissue was then incubated with the appropriate primary antibody (anti-SK3, anti-NCX, anti-THP, or anti-AQP2 ATTO-550) overnight at 4°C (see Table 1). Following wash of primary antibodies with TBS, sections were incubated with secondary antibodies (Cy5 anti-rabbit, fluorescein labeled peanut agglutinin (PNA-FITC), Cy2 anti-sheep, Cy2 anti-mouse) for 3 hrs at room temperature. The tissue sections were mounted on coverslips with VectaShield mounting medium (Vector Laboratories). The sections were then imaged on a Nikon A1 confocal microscope. From 2–3 sections were imaged from each of 3 kidneys to evaluate SK3 expression in each tubular segment. Representative images were selected from each condition reported, as done previously [31], [32], [49]. For CCD, SK3 intensity profiles were obtained for each cell in a x-sectional view using Image J (NIH, version 1.46r) to define a linear profile line through the cell (apical to basal) to obtain maximal intensities across the apical (luminal) and basal (abluminal) membranes and minimal intensities within the cytosol. The intensity profiles were determined for PCs (AQP2 positive) and ICs (AQP2 negative). All cells from 2 x-sections from each kidney were analyzed in an identical manner to provide an adequate number of cells for statistical analysis (37 PCs, 12 ICs).

**Table 1:** Antibodies and markers used for immunohistochemistry.

Antibody	Dilution	Host	Vendor
Anti-SK3, N-terminus	1∶100	Rabbit	Alomone
Anti-AQP2 ATTO-550	1∶200	Rabbit	Alomone
Anti-NCX	1∶500	Mouse	Swant
Anti-THP	1∶1000	Sheep	Millipore
PNA-FITC	1∶1000		Vector

### Isolation & preparation of split-open collecting ducts

Sections of CCD were isolated and prepared from mouse kidney tissue as previously described [32], [50], [51]. In short, mice were sacrificed by CO_2_ treatment and subjected to cervical dislocation. Subsequently, kidneys were dissected and cut into transverse sections (∼1 mm thick); then placed into ice cold PBS (pH 7.4). Medullary-cortical strips of tubules were dissected from the tissue and then individual CCD teased from the strips using watchmaker forceps (sites of bifurcation of the CCD were used to identify CCD segments from upstream connecting tubules). Isolated tubules were moved onto poly-L-lysine coated glass chips and placed in a perfusion chamber mounted on an Eclipse Ti Nikon microscope at room temperature. Tubules were then split-open with two sharpened micropipettes and used within 3 hrs of isolation for membrane potential measurements (see below).

### Fluorescence measurement of membrane potential, Vm

DiSBAC_2_(3) (Invitrogen), a voltage-sensitive fluorescent probe, was used to measure relative changes in cell membrane potential, Vm, of individual cells [52]–[54] in split-opened CCDs on coverslips using high resolution fluorescence imaging [32], [43], [55]. The DiSBAC_2_(3) dye has been widely used in a broad range of cells to report Vm. Unless otherwise noted, cells were bathed in a isotonic modified balanced salt solution (MBSS), containing (in mM): 140 NaCl, 5.4 KCl, 0.5 MgCl_2_, 0.4 MgSO_4_, 3.3 NaHCO_3_, 2 CaCl_2_, 10 Hepes, 5.5 glucose, and pH 7.4. Prior to imaging, cells were loaded with dye by incubation in MBSS containing 100 nM DiSBAC_2_(3) for 30 min at RT in the dark. The coverslips were mounted in a perfusion chamber (see above) on the stage of a high-resolution Nikon Eclipse Ti inverted fluorescence microscope equipped with a Lambda LS Xenon arc lamp illuminator and filter wheel (Sutter Instruments) and a CoolSNAP HQ^2^ cooled CCD camera (Photometrics) as before [32], [55]. Whereupon, DiSBAC_2_(3) was added to all perfusion solutions throughout the experiment. The fluorescence signal (images) was acquired using standard procedures (excitation wavelength = 530 nm and emission wavelength = 580 nm) [52]–[54]. The association of the negatively charged fluorescent probe to the cell membrane is a function of membrane potential. Depolarization of the membrane leads to accumulation of the probe near the cell membrane and is associated with an increase in fluorescence; conversely hyperpolarization of the membrane leads to dispersion of the probe away from the cell membrane and is associated with a decrease in fluorescence. Regions-of-interest, ROIs, were drawn around peripheral membrane areas of individual cells for measurement of fluorescence intensities (one ROI/cell). Correction for background signals was performed by selecting ROIs in regions without cells and subtracting this background fluorescence signal from all cell measurements. All fluorescence measurements were reported as relative fluorescence units (RFU). A High K^+^ solution containing 50 mM KCl (High K^+^ solution: 50 mM KCl substituted for NaCl in MBSS) was used as a standard test for inducing a defined membrane depolarization. For statistical analysis, 7–15 ROIs from each split-open CCD were selected based on the Vm response to application of the High K^+^ solution. Typically from 3–5 CCDs were isolated and used from 2–4 kidneys (1–2 CCDs/kidney) for each treatment group. “n” is representative of the number of cells analyzed for all tubules in each group. Data are presented as a mean value ± SEM.

### Chemicals

The following chemicals were used in this study: GSK101 (GSK1016790A, Santa Cruz Biotechnology) from a stock solution (1 µM) in DMSO; apamin (Apa, Alomone) from a stock solution (1 mM) in PBS; and iberiotoxin (IbTX, Alomone) from a stock solution (0.1 mM) in PBS.

### Statistical Methods

Summary data are given as mean values ± SEM as indicated in the figures. Differences among groups was analyzed with either the *t*-test, when comparing only two groups, or a one-way ANOVA for larger groups followed by the Holm-Sidak *a posteriori* test to define significant differences among groups. The significance level was defined as P<0.05; n is the number of cells assessed in each group.

## Results

### SK3 expression in the mouse kidney

As an initial step toward identifying the expression of SK3 channels in renal tubules, we assayed for both mRNA and protein expression levels using RT_PCR and immunoblotting from whole kidney samples. As shown in Figure 1A, kidney mRNA analysis revealed relatively high levels of SK3 expression similar to that observed for BKα. Immunoblots from whole kidney homogenates, using a well-characterized antibody against mouse SK3 (see Materials and Methods), revealed a prominent band near 90 kD, consistent with expression of SK3 in kidney (Figure 1B) as shown for SK3 in other tissues [56]–[59]. As a negative control, an SK3 blocking peptide was used to verify the specificity of our SK3 antibody which, as shown, abolished the SK3 band (Figure 1B, SK3-BP lane). Hence, both RT-PCR analysis and immunoblots demonstrate prominent SK3 expression in mouse kidney.

### SK3 expression along the nephron

**Figure 2:**
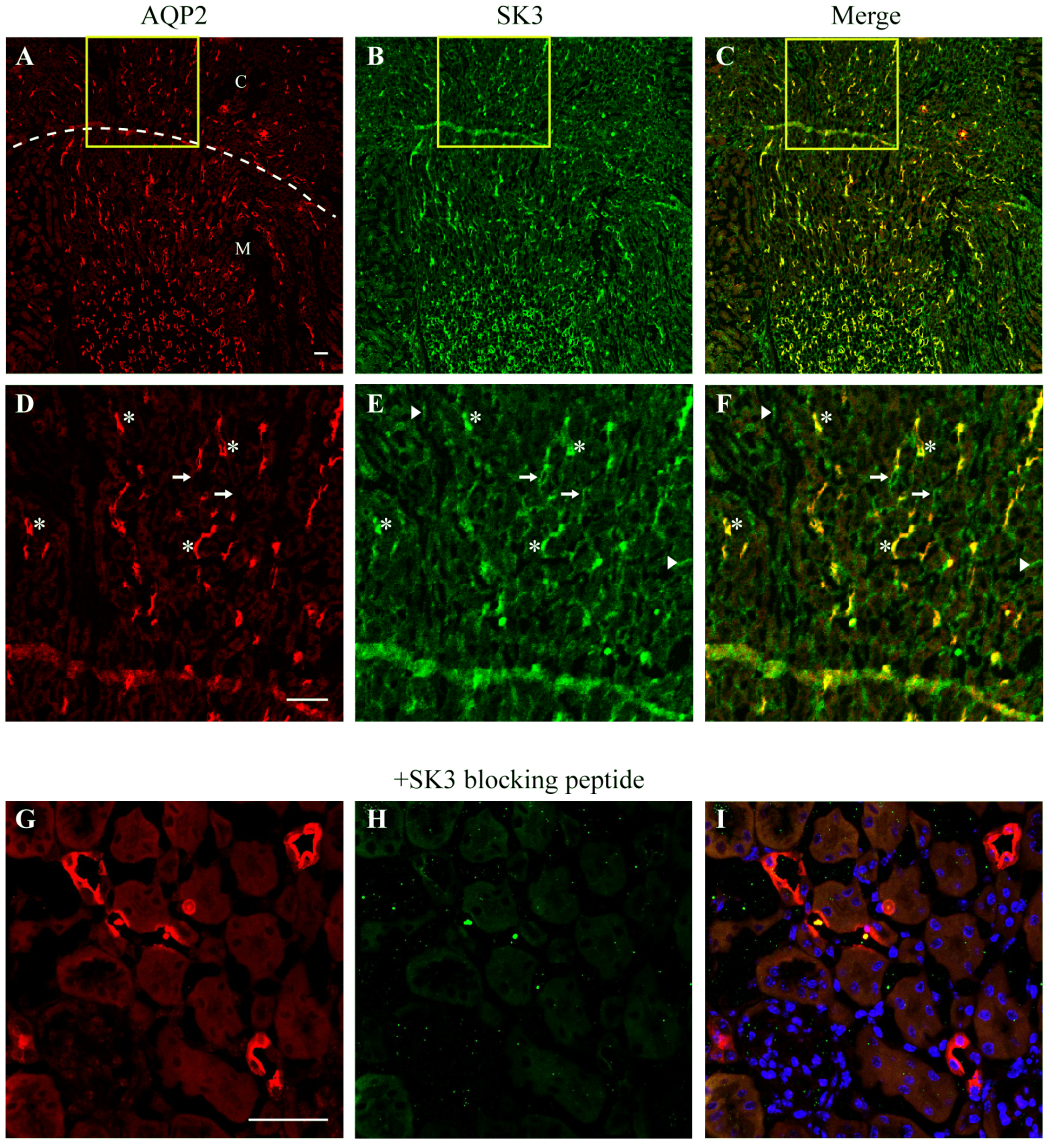
Immunohistochemical staining for SK3 and aquaporin-2 (AQP2) in WT mouse kidney sections. Top Panel (A–C): A low-magnification transverse section (5 µm) of the mouse kidney is shown. Discrete labeling is shown for staining for aquaporin-2 (**A.** AQP2, red), a marker of the collecting ducts, SK3 (**B.** SK3, green), and a merger of both channels (**C.** Merge, yellow-organge for co-localization of AQP2 and SK3). Labeling is apparent for SK3 in both the cortex (label C) and medullary (label M) (dashed line shows cortical-medullary demarcation). **Middle Pannel (D–F):** Magnified view of the yellow inset box from A. SK3 co-localizes with all AQP2-postive tubules as show by the yellow-orange images (F., asterisk). SK3 staining is also apparent in AQP2-negative structures including other tubular structures (F., arrows) and smaller secondary structures (possibly vascular structures, F., arrow heads). **Bottom Panel (G–H):** Magnified view of staining in the presence of SK3 blocking peptide. All SK3 staining is abolished demonstrating specificity of our anti-SK3 antibody. Scale bar is 50 µm.

To identify the sites of expression of SK3 channels in renal tubules, mouse kidney sections (5 µm thick) were immunostained for SK3. We employed Alomone Lab's anti-SK3 antibody directed against the N-terminus as it has been shown to display high specificity for SK3 in a wide range of cell types and tissues (see Materials and Methods). Initial studies included co-staining for Aquaporin 2 (AQP2), a marker of PC cells within the collecting duct system (from CNT through inner medullary collecting duct). Transverse kidney sections revealed substantial binding of anti-SK3 antibody to discrete tubular structures within the cortex and medulla (Figure 2B and 2E). In agreement with our previous findings using M-1 collecting duct cells [43], much of the SK3 staining was located in the collecting ducts, as evidenced by co-localization of SK3 with AQP2 (Figure 2A–2C; 2D–2F, asterisk). In addition, SK3 staining was also apparent in tubular structures which did not show AQP2 expression, reflecting likely expression in other tubule segments (Figure 2D–2F, arrows). The smaller tubule-like structures likely represent SK3 expression in the renal vasculature endothelial cells since most endothelial cells are known sites of SK3 expression (Figure 2D–2F, arrow heads) [60]–[62]. To verify the specificity of our SK3 antibody, immuno-staining studies were also performed in the presence of the SK3 blocking peptide. As shown by the example in Figure 2G–2I, SK3 staining was abolished in the presence of the blocking peptide, demonstrating specificity of our anti-SK3 antibody for the SK3 epitope in the mouse kidney.

In order to further elucidate the sites of expression of the SK3 channel along the distal nephron, kidney sections were co-immunostained for SK3 and selective markers of defined tubule segments (see Table 1). This included antibodies against Tamms Horsfall Protein (THP), a marker of the thick ascending limb, the Na^+^/Ca^2+^ exchanger (NCX), a marker of the distal convoluted tubule and, as above, AQP2, a selective marker of PC cells of the collecting duct system (see Table 1, [63]–[65]).

**Figure 3:**
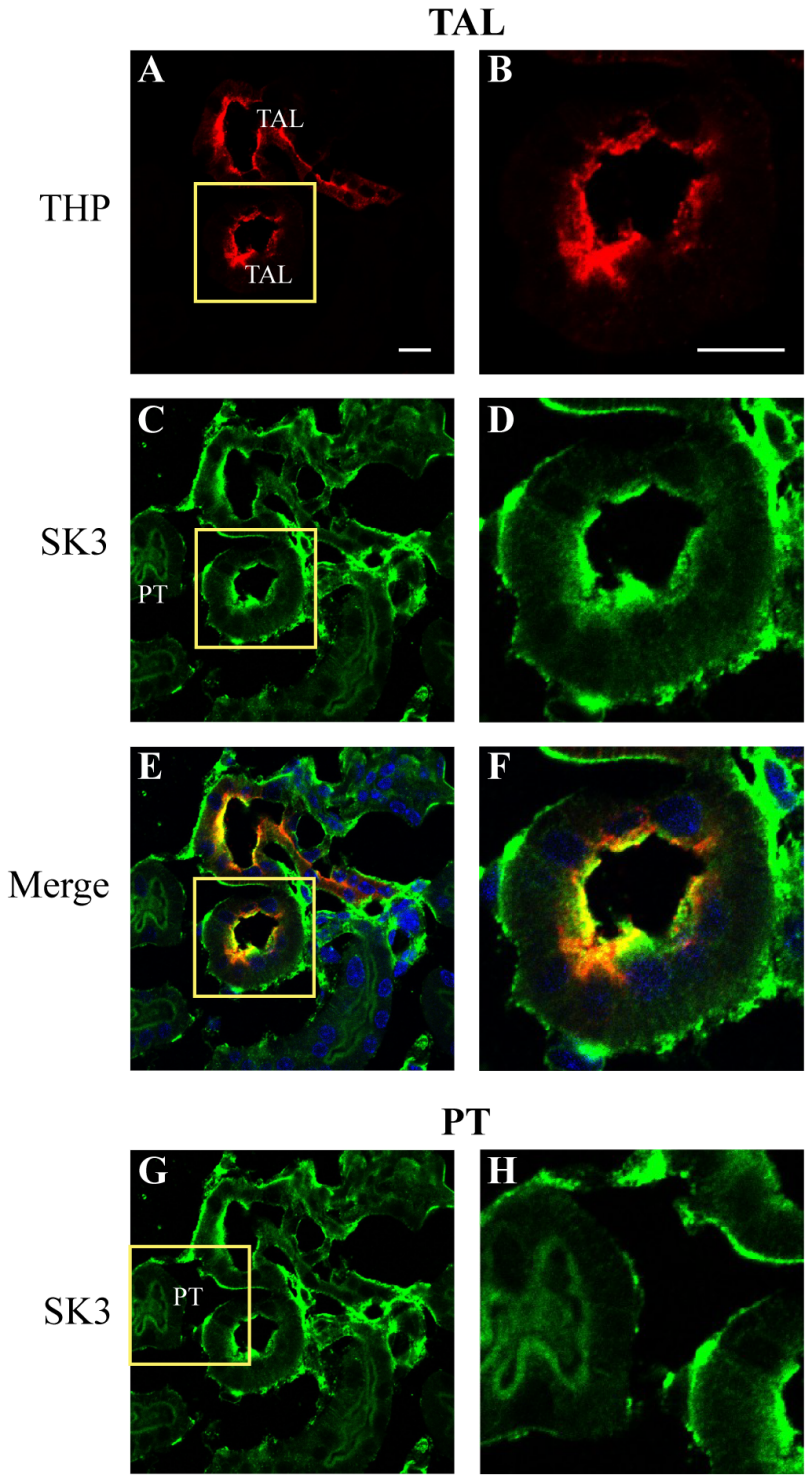
Immunohistochemical staining of SK3 for thick ascending limb tubules. Sagital section (5 µm) of WT mouse kidney showing staining for Tamm-Horsefall protein (THP, red), a marker of TAL cells, and SK3 (green). **Panels A, C, E, and G** are low magnification images showing THP staining of TAL structrues (**A**), SK3 labeling of the same structures (C), and a merged image (E). As shown at higher resolution for one of the tubules (inset from A), THP strongly stains the luminal border of the TAL (**B** and **F**) with SK3 also showing strong labeling of the luminal border and, to a variable degree, the abluminal border (**D** and **F**). The merged image (**F**) clearly identifies SK3 staining in the TAL cells. Panel **H** is a magnified view of a proximal tubule (PT), located left of the TAL in **A**. The PT showed minimal staining for SK3, although light staining was apparent along the luminal brush border. Scale bar is 10 µm.

In thick ascending limb segments (THP-positive) significant SK3 expression was apparent along the luminal cell border (Figure 3C, 3D) with considerable colocalization with THP (Figure 3E–3F). Some SK3 staining was also apparent along the abluminal cell borders, although the intensity of staining was more variable (Figure 3D). The thick ascending limb is a prominent site for ROMK expression which functions to secrete K^+^ into the tubular lumen as part of the K^+^ recycling processes in TAL [25]–[27], [66]. Whether SK3 contributes to this process in stimulated states, i.e. states of elevated [Ca^2+^]_i_, is not currently known, but its expression along the luminal border would be consistent with this view. Figure 3 also shows a section through a proximal tubule which shows minimal SK3 staining, although weak staining is apparent along the luminal brush border of proximal tubule cells (Figure 3G and 3H, PT label).

**Figure 4:**
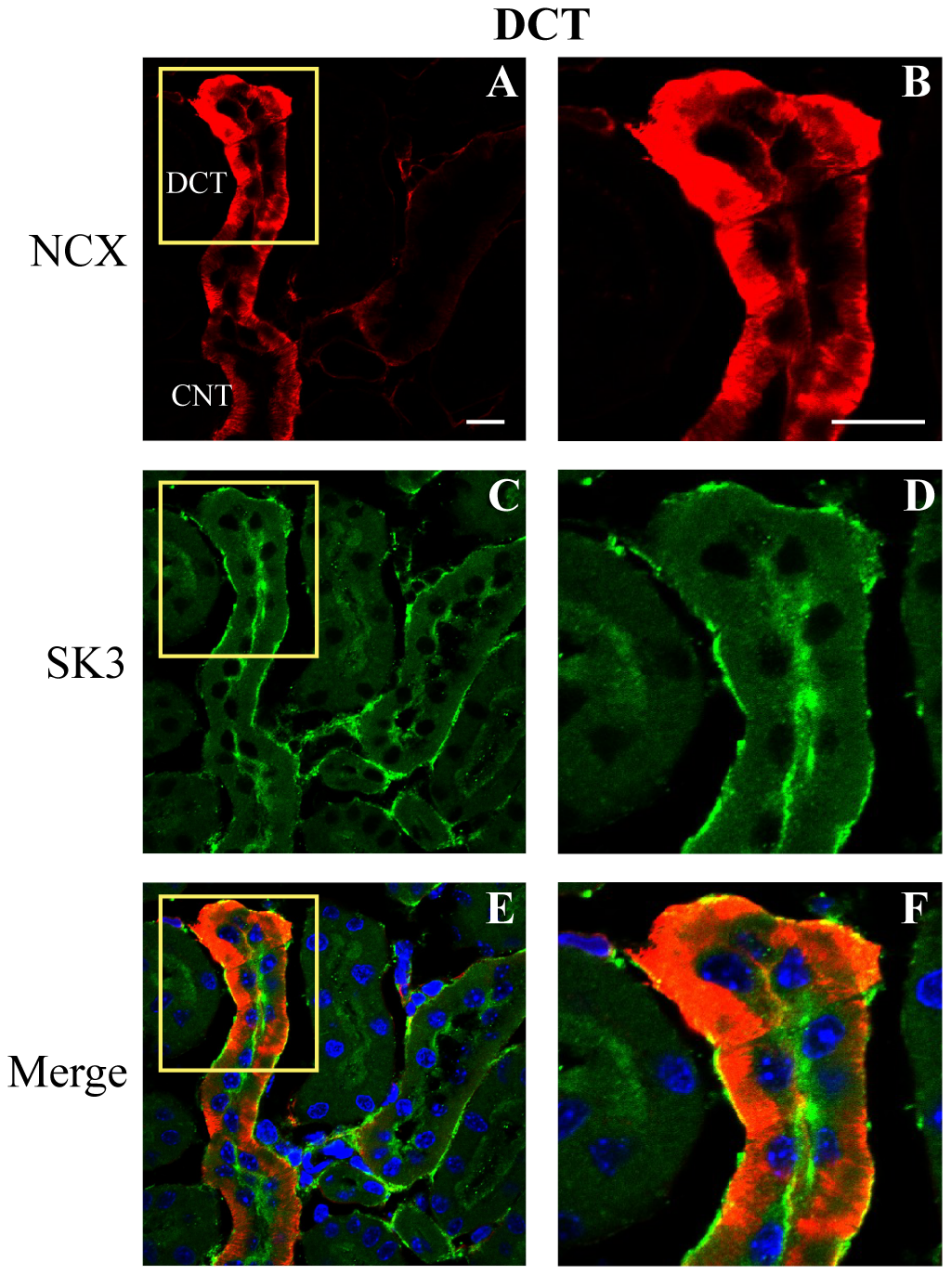
Immunohistochemical staining of SK3 in the distal convoluted tubule (DCT). Mouse (WT) kidney section (5 µm) showing staining for the sodium-calcium exchanger (NCX, red), a marker of DCT, especially the later portion (DCT2), and SK3 (green). The heavy NCX staining of the upper portion of the tubule in **Panel A** (within yellow inset box) is consistent with the DCT2 segment with the weaker, more basolateral staining in the lower half of the tubule indicating this is the connecting tubule (CNT) (see text for details). Higher resolution image of the DCT2 (**D**) shows strong staining of SK3 along the luminal border with more variable, weaker staining along the abluminal border. The merged image clearly identifies SK3 staining of the DCT (**F**). In the CNT segment (**A.**, labeled CNT), SK3 staining was also apparent along the luminal border with abluminal staining appearing weaker. Scale bar is 10 µm.

SK3 staining was also prominent along the distal convoluted tubule (DCT) segments, a site that is noted for ROMK expression and modest K^+^ secretion. The tubule segments in the upper portion of Figure 4 show prominent cytoplasmic and abluminal staining by anti-NCX, a marker of the DCT, particularly of the later segment, the DCT2 [64]. As evidenced in the detailed image (Figure 4C and 4D), SK3 is highly expressed along the apical border of the cells, but again, with staining apparent along the abluminal border of some cells. The tubular structure in Figure 4A and 4B also demonstrates that the lower half of the NCX-positive tubule in the figure shows a marked reduction in NCX staining with a shift in staining towards the abluminal membrane. This is characteristic of the transition from DCT2 to the CNT [64]. Hence, the lower half of the tubule is likely representative of the CNT. SK3 staining of the CNT segment shows more prominent apical membrane staining with greatly reduced abluminal staining (Figure 4C, lower half, CNT label), similar to that observed for the CCD as detailed below. Again, since ROMK is also expressed at the luminal border of distal tubule segments to effect K^+^ secretion, it may be that SK3 contributes to this process under stimulated states.

### SK3 expression and function in cortical collecting duct

**Figure 5:**
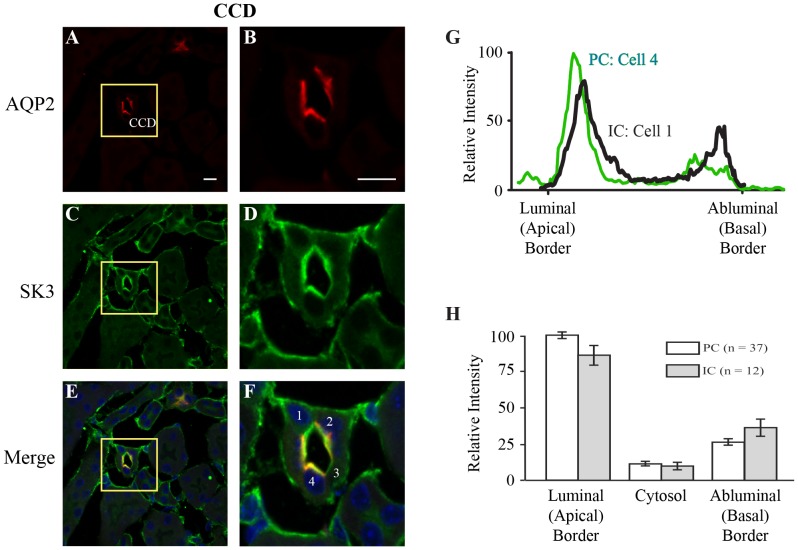
Immunohistochemical staining of SK3 in the collecting duct. Section (5 µm) from WT mouse kidney showing staining for AQP2 (red), a marker of PCs in collecting duct, and SK3 (green). **Panels A, C, and E** are low magnification views of a cross-section through a CCD identified by AQP2 staining. **Panels B, D, and F** represent a magnified view of the inset area from **A** (yellow inset box). **Panel B** shows strong AQP2 staining along the luminal border of PCs (5–6 cells), but not of the ICs (2 cells without staining). As shown in **D** and **F**, strong staining of SK3 is evident along the luminal border of all cells, both PCs and ICs. Variable, but weak staining, is also apparent along the abluminal border of some cells. However, the staining is most pronounced along the luminal border for both PCs and ICs, although typically stronger in PCs, as indicated by the SK3 fluorescence line intensity profiles across (luminal to abluminal direction) two cells identified as PC and IC (**Panel G**). **H**. Relative mean intensity profiles (± SEM) across the cells from all sections showing the maximal values across the luminal border (Apical) and abluminal border (Basal) and the minimal values within the cytoplasm (Cytosol). The mean values are given for both PCs (n = 37) and ICs (n = 12) from all sections analyzed. The maximal luminal intensity is much greater than the abluminal intensity (*P<0.02) indicating dominant expression at the luminal border. Scale bar is 10 µm.

Kidney sections and isolated CCD tubules were used to identify the expression pattern of SK3 in the mouse collecting duct. Staining of kidney sections with SK3 and AQP2 revealed expression of SK3 channels along the entire collecting duct (Figure 2 and 5). Figures 5B, 5D, and 5F shows immunostaining results in a cross section of a CCD. AQP2 staining is evident along the luminal border of 5–6 cells, identifying these cells as PCs, while two other cells did not stain for AQP2, identifying these as ICs (Figure 5B and 5F). Prominent SK3 staining, however, was apparent along the luminal border of all cells of the CCD (Figure 5D). SK3 expression in PCs was characterized by relatively high SK3 staining of the apical membrane and subapical regions with weak or variable staining along the abluminal border (Figure 5E). Similarly, most ICs showed significant levels of SK3 expression along the apical membrane/subapical regions with minimal or variable staining of the abluminal border (Figure 5E). Separate immunostaining for SK3 alone, without AQP2 immunostaining, showed similar SK3 localization (data not shown). A line intensity profile of SK3 immunofluorescence along the luminal-to-abluminal axis of both PCs and ICs indicates prominent SK3 expression along the luminal border in all cells of the CCD with the PC typically showing modestly higher levels of staining as shown by the representative example for a PC and IC in Figure 5G (obtained from the cells identified in Figure 5F). Indeed, the relative maximal fluorescence intensity across the luminal and abluminal borders averaged 100±2.4 and 24.4±2.2 (n = 37) relative units in PCs (P<0.02) and 87.5±6.9 and 36.7±5.9 (n = 12) relative units in ICs (P<0.02), respectively, confirming the dominant expression of SK3 at the luminal border of both PCs and ICs (Figure 5H).

**Figure 6:**
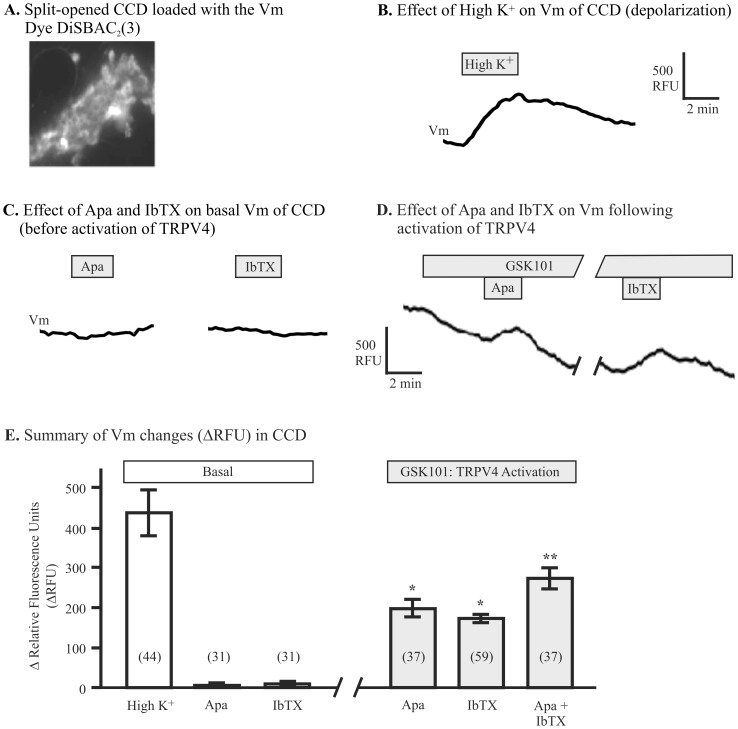
Effect of TRPV4-mediated activation of SK3 channels on membrane potential, Vm. **A.** Fluorescence image of a split-open CCD loaded with the voltage-sensitive fluorescence dye, DiSBAC_2_(3), showing loading of all cells. The fluorescence intensity is an index of Vm and is presented as relative fluorescence units (RFU). **B.** Effect of 50 mM K^+^ (High K^+^) application on Vm of CCD cells showing the expected membrane depolarization (increased RFU). **C.** Effect of 300 nM apamin or 50 nM IbTX application on Vm in basal conditions showing little or no effect of either apamin (Apa) or IbTX in the basal state (TRPV4 not activated). **D.** Effect of TRPV4 activation with GSK101 (50 nM) leading to membrane hyperpolarization of Vm (decreased RFU), as expected for SK3 and BK activation. Subsequent application of either 300 nM apamin or 50 nM IbTX now induce a marked depolarization of Vm (increased RFU) demonstrating inhibition of SK3 and BK, respectively. **E.** Summary graph showing mean changes in Vm in basal conditions upon addition of High K^+^ (High K^+^, n = 44 cells), 300 nM apamin, or 50 nM IbTX (Left panel, Basal). Right panel (GSK101: TRPV4 Activation) shows the results after activation of TRPV4 (Ca^2+^ influx). Both apamin and IbTX now bring about a significant depolarization of Vm (*P<0.01 compared to Basal). The combine addition of both apamin and IbTX (Apa + IbTX) displays an enhanced depolarization compared to addition of apamin or IbTX alone (**P<0.01). The number in parentheses is the number of cells for each group (n).

Finally, the functional activity of SK3 channels was investigated in split-open CCD tubules with the voltage-sensitive fluorescent probe, DiSBAC_2_(3), as done by others [52]–[54], [67]. This dye has been widely used to reproducibly report Vm under both depolarizing and hyperpolarizing conditions in a broad range of cells. Changes in the fluorescence intensity of DiSBAC_2_(3) reflects changes in Vm where an increase in fluorescence intensity (Relative Fluorescence Units, RFU) correlates with a depolarization of Vm and a decrease in fluorescence intensity with a hyperpolarization of Vm. A 30-min loading period with the dye (100 nM) provided an excellent fluorescence signal over background (Figure 6A). As a control test and an index of cell viability, cells incubated with the DiSBAC_2_(3) fluorescent probe were also briefly exposed to a High K^+^ solution (50 mM K^+^, Figure 6B) to depolarize the cell membrane. As shown in the representative trace, exposure to a High K^+^ solution led to an increase in the fluorescence intensity at the cell membrane reflecting the expected depolarization of Vm.

To determine if activation of the SK3 channel contributed to Vm, the effect of apamin, a selective SK channel antagonist, was tested along with the BK channel selective antagonist, iberiotoxin (IbTX). Under basal [Ca^2+^]_i_ conditions, addition of apamin (300 nM) or IbTX (50 nM) had little or no effect on Vm (Figure 6C) with RFUs changing by 1.0±3.1 (n = 31) and 7.9±3.5 (n = 31) RFUs, respectively, although a few cells, but not all, responded to addition of IbTX. The data support the view that both channels are relatively quiescent under basal conditions. In contrast, following activation of TRPV4 using the selective agonist, GSK101 (50 nM), thereby inducing Ca^2+^ influx and a rise in [Ca^2+^]_i_ as shown before (see [32], [43], [55]), Vm typically hyperpolarized, reflecting activation of Ca^2+^-activated K^+^ channels (Figure 6D). Indeed, addition of either apamin or IbTX in the presence of GSK101 now induced a significant membrane depolarization (increased fluorescence) as shown by the representative example in Figure 6D. Data from all studies is summarized in Figure 6E. As shown, the mean fluorescence intensity increased by 197±21.7 RFUs upon addition of apamin (Apa, n = 37) and 172±9.5 RFUs upon addition of IbTX (n = 59). The combined addition of apamin and IbTX brought about a greater increase in RFUs, averaging 276±26.6 RFUs (n = 37). The increase in RFUs was significantly greater upon addition of both inhibitors relative to either inhibitor alone (P<0.02). Hence, inhibition of SK3 induced a membrane depolarization that was similar in magnitude as that observed for inhibition of the BK channel, indicating both channels are activated by TRPV4-mediated Ca^2+^ influx. The two channel types appear to operate in parallel since inhibition of both SK3 and BK induced a further depolarization in the CCD cells than inhibition of either channel alone. The results of these studies are consistent with that observed for SK3 and BK expression in M-1 CCD cell line where hypotonicity-induced cell swelling induced Ca^2+^ influx with the subsequent activation of both SK3 and BK [43]. Hence, these data demonstrate that functional SK3 channels are expressed in mouse kidney distal nephron and collecting duct cells and that TRPV4-mediated Ca^2+^ influx can gate these channels as shown here for collecting duct cells.

## Discussion

In the present study we assessed the potential functional expression of the Ca^2+^-activated, small conductance, SK3 K^+^ channel in the nephron. Here we demonstrate for the first time that the SK3 protein is expressed in the mouse kidney with the highest levels of expression apparent in tubular segments of the distal nephron and collecting ducts. Using segment-specific markers we show that SK3 is expressed in the thick ascending limb, the distal convoluted tubule, the connecting tubule and the entire collecting duct system. Cellular sites of expression show dominant luminal membrane staining, although significant basolateral (abluminal) membrane staining is apparent in some cells, especially in the earlier segments of the distal nephron. Since we also show that the channel is functional, being activated by Ca^2+^ influx and leading to hyperpolarization of Vm, it follows that SK3 may play a key role in Ca^2+^-dependent regulation of membrane potential and K^+^ transport in these distal nephron and collecting duct segments (see below). Indeed, the sites of SK3 expression largely mirror those for the Ca^2+^-independent ROMK channel (see [25]). Since ROMK is a known key channel effector of K^+^ secretion in these segments, it is likely that SK3 shares some of this K^+^ secretory function, especially under stimulated states with elevated [Ca^2+^]_i_ levels.

We extended our analysis of SK3 expression and function in the CCD since this is a major site for regulating K^+^ secretion in the kidney [23], [28], [68]. Using AQP2 as a marker of PCs in the CCD [63], our immunohistochemical staining shows apparent high levels of expression of SK3 in both the PC (AQP2 positive) and the IC (AQP2 negative) (Figure 5). This expression is dominant at the apical membrane and subapical regions of the cells, an expected pose for a K^+^ secretory channel. Using freshly isolated split-opened mouse CCD, we measured changes in the membrane electrical potential, Vm, using the fluorescence dye DiSBAC_2_(3), as done by many other groups [52]–[54], [67]. Addition of the SK selective channel blocker, apamin, had little or no effect on Vm under basal conditions. However, after activation of TRPV4 to induced Ca^2+^ influx, leading to membrane hyperpolarization as expected for activation of K^+^ channels, apamin now induced a significant depolarization of Vm demonstrating functional SK3 channels in the CCD following induction of TRPV4-mediated Ca^2+^ influx (Figure 6). The depolarization response was subsequently verified by blockade of the BK channel, a well-characterized Ca^2+^-dependent K^+^ channel in CCD [5], [26], [69], were addition of the BK selective antagonist, IbTX, was shown to have a similar depolarizating effect on Vm as that observed for blockade of SK3 channels (Figure 6). These studies demonstrate that both SK3 and BK are functional in the CCD and both are activated by TRPV4-mediated Ca^2+^ influx. Hence, SK3 must play a key role in regulating the Vm in CCD during states of induced Ca^2+^ influx. Indeed, this view is supported by our recent studies in mouse M-1 collecting duct cells where we show that modest Ca^2+^ influx not only activates SK3, but that the associated membrane hyperpolarization with SK3 activation, in turn, serves to further enhance Ca^2+^ influx through non-voltage-activated TRP channels [43]. Hence, this same phenomenon leading to a positive coupling between Vm and Ca^2+^ influx will likely be at play for SK3 in the intact CCD. It remains for future studies to fully characterize the SK3 channel properties and to define the extent that it underlies control of Vm and K^+^ secretion in the CCD.

Activation of SK3 in the mouse CCD by elevation of Ca^2+^ influx would appear to be, at least in part, under the control of the Ca^2+^-permeable TRPV4 channel. We have previously shown that TRPV4 is highly expressed in the mouse kidney (the source of our homology-based TRPV4 clone [70]),which we demonstrated is localized to the entire collecting duct including the connecting tubule and CCD [31], [32]. Using the selective TRPV4 agonist, GSK101 (GSK1016790A, [71]), we now show that TRPV4-mediated Ca^2+^ influx appears to be a key regulator of both SK3 and BK activity in CCD (Figure 6D, 6E). These findings are consistent with our previous studies in mouse M-1 CCD cells where we demonstrated that both SK3 and BK are closely regulated by TRPV4-mediated Ca^2+^ influx following either activation of TRPV4 by osmomechanical stimulation (hypotonicity) or direct activation by addition of GSK101 [43]. Furhter, while the Ca^2+^ dependence of flow-induced K^+^ secretion in CCD is well known [30], the role of TRPV4 in this process is developing where recent studies in TRPV4-deficient mice show that both flow-induced Ca^2+^ signaling [32] and flow-induced K^+^ secretion by the CCD are markedly blunted [28], as heretofore noted. Hence, TRPV4 would appear to play a central role in regulating the Ca^2+^-dependent K^+^ channels and, in turn, Vm and K^+^ secretion in the CCD.

While the precise role of TRPV4 in control of Ca^2+^-activated K^+^ channels in CCD is still emerging, a similar close association between TRPV4 and SK3/IK channels has recently been shown in vascular endothelial cells. This association appears to be responsible, at least in part, for the “generation” of the endothelial-derived hyperpolarizing factor (EDHF) which is a key signaling factor leading to vascular vasodilations and a reduction in blood pressure (see [72]–[74]). It has been shown that TRPV4, SK3 and IK are expressed in a wide range of endothelial cells [9], [60], [75], [76]. Recent studies have shown that activation of endothelial cell TRPV4 channels or SK3/IK channels via fluid flow or by muscarinic receptor activation (acetylcholine), activates various pathways including EDHF-mediated signaling leading to hyperpolarization of the endothelial cell. This response appears to be communicated through myoendothelial gap junctions to hyperpolarize the underlying smooth muscle cells which, in turn, contributes to relaxation of the vessel and vasodilation [60]. In vessels from animals either deficient in TRPV4 or SK3/IK, or where either TRPV4 or SK3/IK have been blocked pharmacologically, the effect of stimulation by flow or acetylcholine administration on membrane hyperpolarization and vessel dilation is markedly attenuated, largely abolishing the EDHF signaling and membrane hyperpolarization [9], [60], [76], [77]. The activation of TRPV4 is a key component in this response since the associated Ca^2+^ influx will regulate activation of SK3 and IK and, in turn, Vm, a response similar to what we report here for SK3 and BK in the CCD.

In other studies of vascular tissue it has recently been shown that TRPV4 and SK3 associate with each other in the endothelial cell plasma membrane [78] and that small, cooperative, complexes of TRPV4 channels (four channels per complex) exist where activation of just a few TRPV4 channels is sufficient to fully activate SK3 [73], [79]. Similarly, such a close association has also been proposed for the underlying smooth muscle cells where TRPV4, or a TRPC1-TRPV4 complex [67], closely associates with the BK channel, possibly as another signaling complex which, in turn, contributes to hyperpolarization of Vm and smooth muscle relaxation as part of the vasodilatory response [67], [80]. Whether such a close association of TRPV4 with either SK3 or BK into a signaling complex exists in the CCD is currently not known, but would appear highly likely given that the same channels (TRPV4, SK3, and BK) appear to underlie Ca^2+^ influx and regulation of Vm in the CCD. Nonetheless, it remains for future studies to define the nature of the association between TRPV4 and SK3/BK in the CCD and to identify potential microdomain and/or macromolecular structures that may give rise to the functional coupling among the channels.

Does SK3 play a role in K^+^ secretion and, hence, K^+^ homeostasis? Indeed, with the observed expression of SK3 at the apical cell membrane in distal nephron and collecting duct segments, it is likely that SK3 is a key contributor to K^+^ secretion under stimulated states that give rise to elevated cytosolic Ca^2+^ levels. It is well known that basal K^+^ secretion in the CNT and CCD, the main sites for K^+^ secretion, is attributable to the calcium-insensitive ROMK channels [5], [25]–[27]. However, under states of enhanced K^+^ secretion, such as with elevated fluid delivery to the distal tubule, the Ca^2+^-activated BK channel has been shown to be a key contributor to the enhanced K^+^ flux [22], [28], [68]. It is also known that elevated fluid delivery stimulates Ca^2+^ influx, which we and others have shown arises from flow-induced TRPV4 activation [28], [30]–[32], as heretofore noted, leading to activation of the BK channel and the enhanced K^+^ secretion. While blockade of BK, or use of animals deficient in BK, has shown that a major fraction of the flow-induced K^+^ secretion is dependent upon BK, it has also recently been shown that the elevated flow rates stimulate ATP release from distal tubule cells [40] which, in turn, inhibits ROMK and the basal K^+^ secretion [42]. Hence, this begs the question as to what other K^+^ channel may be contributing to K^+^ secretion under these stimulated states. SK3 is postured to fit this role since the TRPV4-mediated Ca^2+^ influx will activate the channel in native CCD as shown in the current study. We also speculate that since SK3 has a much higher Ca^2+^ affinity than BK (see Introduction) that SK3 may actually be the first channel activated under states of elevated flow, although this remains to be directly assessed.

Finally, SK3 is also likely to play a role in other K^+^-channel dependent phenomena such as cell volume regulation. Cell swelling of many epithelial cells leads to enhanced Ca^2+^ influx and activation of Ca^2+^-activated K^+^ channels to induce K^+^ efflux, along with Cl^−^, leading to solute loss and regulatory volume decrease (see reviews [81]–[84]). Indeed, we demonstrated a few years ago that, in native, isolated perfused, CCDs, induction of PCs to swell by current transfer techniques was immediately followed by cell volume regulation back to control volume states, a process that was dependent upon the Ba^2+^-sensitive apical K^+^ channels [85] that would include ROMK and all K_Ca_ channels. Others have shown that CCD cells in culture undergo regulatory volume decrease upon cell swelling [86] and that cell swelling activates TRP channels to induce Ca^2+^ influx in collecting duct cells [43], [86], [87]. Since we have also shown that, in M-1 cells, cell swelling activates TRPV4 and Ca^2+^ influx which, in turn, leads to activation of both BK and SK3, it seems reasonable to conclude that swelling states in native CCD and CNT, at least for PCs, leads to activation of both BK and SK3 to effect cell volume regulation. However, the precise role of BK and SK3, or other Ca^2+^-activated K^+^ channels, in this process remains to be fully elucidated in future studies.

In summary, the current study provides evidence for the expression of functional SK3 channels along the mouse distal nephron with high levels of expression in the distal tubule and the entire collecting duct. In CCD, SK3 is expressed in both PCs and ICs, with prominent localization at the apical membrane and subapical regions of the cell. The SK3 channel is activated upon stimulation of TRPV4 and elevation of intracellular Ca^2+^ levels and, as such, likely plays a key role, along with other Ca^2+^-activated K^+^ channels, in regulating both membrane potential and K^+^ secretion during states of elevated flow rates or cell volume regulation. It remains to be determined whether these Ca^2+^-activated K^+^ channels and Ca^2+^-permeable TRP channels function as independent entities or, more likely, associate into microdomains as macromolecular signalingplexes to bring about a coordinated control of channel functions in specific cell types of the distal nephron and collecting ducts.
