# Correction: Emerging Role of the Calcium-Activated, Small Conductance, SK3 K^+^ Channel in Distal Tubule Function: Regulation by TRPV4

**DOI:** 10.1371/journal.pone.0156368

**Published:** 2016-05-19

**Authors:** Jonathan Berrout, Mykola Mamenko, Oleg L. Zaika, Lihe Chen, Wenzheng Zhang, Oleh Pochynyuk, Roger G. O'Neil

The fifth author’s name is spelled incorrectly. The correct name is: Wenzheng Zhang. The correct citation is: Berrout J, Mamenko M, Zaika OL, Chen L, Zhang W, Pochynyuk O, et al. (2014) Emerging Role of the Calcium-Activated, Small Conductance, SK3 K^+^ Channel in Distal Tubule Function: Regulation by TRPV4. PLoS ONE 9(4): e95149. doi:10.1371/journal.pone.0095149
